# The Role of Alexithymia in Social Withdrawal during Adolescence: A Case–Control Study

**DOI:** 10.3390/children8020165

**Published:** 2021-02-22

**Authors:** Sara Iannattone, Marina Miscioscia, Alessia Raffagnato, Michela Gatta

**Affiliations:** Child and Adolescent Neuropsychiatry Unit, Department of Women’s and Children’s Health, Padua University Hospital, 35128 Padua, Italy; marina.miscioscia@unipd.it (M.M.); alessiaraffagnato@gmail.com (A.R.); michela.gatta@unipd.it (M.G.)

**Keywords:** social withdrawal, alexithymia, adolescence, psychological disorders, anxiety, depression, social problems

## Abstract

Although social withdrawal is becoming increasingly common among adolescents, there is still no consensus on its definition from the diagnostic and psychopathological standpoints. So far, research has focused mainly on social withdrawal as a symptom of specific diagnostic categories, such as depression, social phobia, or anxiety disorders, or in the setting of dependence or personality disorders. Few studies have dealt with social withdrawal in terms of its syndromic significance, also considering aspects of emotion control, such as alexithymia. The present case-control study aimed to further investigate the issue of social withdrawal, and try to clarify the part played by alexithymia in a sample of Italian adolescents diagnosed with psychological disorders (*n* = 80; Average Age_g_ = 15.2 years, SD = 1.49). Our patients with social withdrawal (cases) scored significantly higher than those without this type of behavior (controls) in every domain of alexithymia investigated, using the Toronto Alexithymia Scale (TAS-20) and with the scales in the Youth Self-Report (YSR) regarding internalizing problems, anxiety–depression, social problems, and total problems. Internalizing problems and total levels of alexithymia also emerged as predictors of social withdrawal. These variables may therefore precede and predispose adolescents to social withdrawal, while social problems may develop as a consequence of the latter.

## 1. Introduction

Extreme social withdrawal, also known as “hikikomori syndrome”, was first systematically conceptualized at the end of the 1990s by the Japanese psychiatrist Saito [1]. The term was used to refer to individuals who withdraw completely from society for at least six months, refusing to engage in any activities and social relationships. Their circadian rhythm is disrupted, and they may become violent with members of their own family. Such behavior cannot be attributed to other disorders [2].

Hikikomori was initially considered a culture-bound syndrome associated with the features of Japanese society [3,4]. However, when the experiences of these socially-withdrawn young people in Japan attracted more interest, similar cases emerged in other parts of the world too, and it became clear that the phenomenon is not only associated with eastern cultural factors. There are currently no precise data available on the prevalence of social withdrawal in the world’s population.

In Italy, the topic of social withdrawal in adolescence is relatively new, and not enough studies have been conducted to ascertain its statistical prevalence. As reported by the Agenzia Nazionale Stampa Associata (ANSA), in 2018 it was estimated that approximately 100,000 young people in Italy between 13 and 20 years old were socially withdrawn [5]. A study conducted in the same year by the regional school board for Emilia-Romagna on 687 schools found that 21% of them reported cases of pupils who had stopped attending school. In all, 346 cases had been reported to the territorial services, and the majority (59%) of them involved adolescents between 13 and 16 years old [6]. An epidemiological study conducted by a mental health service for children and adolescents run by a local public health unit in Arezzo, central Italy, found that—leaving aside those who were ill—the number of students failing to attend school for more than 40 days accounted for 1% of the school population, with a slight prevalence of males [7].

Generally speaking, several authors have emphasized how difficult it is to conduct cross-cultural studies on social withdrawal, because of the variety of ways in which it can become manifest and be interpreted in different cultures [8,9,10]. For this reason, it is hard to find an unequivocal and shared definition of it in the literature. Some consider it synonymous with hikikomori syndrome, while others use it as an umbrella term covering a vast array of emotions, motives, and behavior associated with the rejection of social interactions [11,12]. According to Asendorpf [13,14], social withdrawal is a multidimensional construct, since it can derive from three different motivations: shyness, peer avoidance, and unsociability. Shyness is a temperamental trait that prevents children and adolescents from interfacing with peers. Shy individuals experience an approach-avoidance conflict because their underlying desire for interaction is inhibited by fear and anxiety. Peer avoidance is the result of need of solitude and avoidance of all the social situations due to fear of judgment. Unsociability, instead, is a characteristic of children and adolescents who are happy alone and are not interested in interacting with peers.

### 1.1. Social Withdrawal in Adolescence: Risk Factor, Symptom, or Clinical Syndrome?

There is currently no consensus on how social withdrawal should be clinically diagnosed. Many psychologists and psychiatrists would agree that the condition of extreme social withdrawal represents a genuine syndrome [7], though it is hard to classify nosographically as a separate entity. In fact, the Diagnostic and Statistical Manual of Mental Disorders fourth edition (DSM-IV-TR) [15] included hikikomori among the cultural syndromes, but then the fifth edition of the Manual (DSM-5) [16] omitted it. The International Classification of Diseases eleventh edition (ICD-11) [17] includes social withdrawal among the “symptoms or signs involving appearance or behavior” in the category of “symptoms, signs or clinical findings not elsewhere classified”, whereas this entity had not been envisaged in the ICD-10 [18].

In the literature on developmental age, social withdrawal has been alternately conceived as a risk factor for the onset of psychological disorders, or as a symptom thereof. Because social withdrawal is reportedly persistent during periods of transition, in the various stages of childhood and in the passage from childhood to adolescence [19], it has been interpreted as an early risk factor for the onset of full-blown psychopathological conditions. Some researchers have found that social withdrawal in developmental age often precedes the onset of various affective–relational problems, including rejection by peers, poor social competence, internalizing problems like anxiety and depression, and loneliness [20,21,22,23]. Rubin and Mills proposed a theoretical model that connects an individual’s social withdrawal (intended as a tendency to remain on the margins of their group) with difficult experiences with their peers, as well as the onset of internalizing problems [24]. These authors suggest that withdrawal behavior at school exposes adolescents to a greater risk of problems with their classmates (rejection, exclusion, victimization). This in turn exacerbates their internalizing symptoms (anxiety, depression, loneliness), further increasing their propensity to become socially withdrawn, and generating a vicious circle. Nevertheless, the relationship between social withdrawal and social problems is still discussed in the literature, because it seems to be unclear whether the former represents a risk factor for the latter or vice versa. For example, according to a research by Oh et al. [25], negative peer relationships (including victimization and rejection) and friendlessness may exacerbate social withdrawal trajectories during childhood and adolescence. Moreover, Spiniello et al. [26] considered social problems as distinctive of the stage before social withdrawal in adolescence. In fact, relational difficulties and lack of interest in interacting with others are typical behaviors that anticipate social withdrawal itself.

Regarding social withdrawal as a symptom of psychiatric disorders, various studies have shown that the diagnoses most often presenting in comorbidity or confused with it are autism, selective mutism, psychotic disorders, personality disorders, mood disorders, anxiety, social phobia, and internet dependence [11,27,28,29]. That said, there have been reports of socially-withdrawn adolescents not meeting any of the criteria for these known psychopathological conditions [8]. Teo and Gaw have consequently recommended including severe social withdrawal as a mental disorder in the DSM [30]. They say it has particular clinical characteristics that distinguish it from the psychiatric problems with which it is often associated (i.e., internalizing disorders, internet dependence, psychotic disorders), but do not enable it to be unequivocally and conclusively defined as a separate disorder.

Suwa and Suzuki have offered a possible solution in the debate over whether social withdrawal is a symptom or a syndrome by distinguishing between primary and secondary social withdrawal [31]. In addition to the inability to be part of and adapt to society, primary social withdrawal also involves a weak self-image that adolescents try to protect with their avoidance behavior. Secondary social withdrawal would instead be one of the symptoms of a known psychopathological condition [32].

### 1.2. Social Withdrawal and Alexithymia in Adolescence

Alexithymia is characterized by the inability to identify and communicate one’s own emotions [33]. Several studies have shown that it is not a condition secondary to situations of stress, but a personality trait that frequently lies behind the onset of psychiatric disorders [34,35,36]. Failure to develop an adequate capacity for emotion control in childhood prevents people from dealing adequately with difficult situations, prompting the emergence of negative feelings that can then impair their mental health.

Referring more specifically to psychological disorders in adolescence, alexithymia has been associated particularly with internalizing disorders [36,37,38,39], eating disorders [40,41], social problems [42], self-harming [43,44], internet dependence [45], borderline personality disorder [46], substance dependence [47], and primary headache [48,49]. In short, studies have placed alexithymia in relation to disorders frequently found in comorbidity with social withdrawal, such as internet dependence, internalizing disorders, and social problems. There is still a severe shortage of published research directly investigating the possible association between social withdrawal and alexithymia, however. One such study was conducted by Frankova, starting from an analysis of the various psychological and psychopathological characteristics of primary and secondary social withdrawal, including alexithymia, comparing groups with the two conditions, and with a healthy control group [29]. Although both the socially-withdrawn groups were less able to identify and verbalize their emotions than the controls, the group with primary social withdrawal showed higher levels of alexithymia than the group with secondary social withdrawal.

Hattori focused instead on the presence of general emotional problems in socially-withdrawn adolescents [2]. The author suggested that these individuals tend to repress their authentic emotions and personality in an effort to adapt to emotionally dysfunctional parents. They create a false identity to protect themselves against other people’s negative opinions of them.

Honkalampi et al. [42] analyzed the correlations between scores obtained on the Toronto Alexithymia Scale-20 (TAS-20) [50] and the Youth Self-Report (YSR) 11–18 [51] scales in a sample of adolescents from the general population. They found the difficulty describing feelings (DDF) factor on the TAS-20 to be more strongly associated with the internalizing problems and withdrawal scales on the YSR. Nevertheless, this study was not specifically focused on the relationship between social withdrawal and emotional difficulties, since it aimed to underline the different psychopathological outcomes linked to alexithymia.

Difficulty describing feelings was also identified in socially-withdrawn adolescents in the clinical experience reported by Piotti, who found these patients unable to find the words to express their experiences of suffering [52].

Other published studies have not associated alexithymia directly with social withdrawal, but have investigated the possible link between difficulties with managing emotions and certain phenomena often encountered in situations of self-isolation, such as interpersonal problems [53], loneliness [54], and shyness [55]. It therefore seems interesting, from both the clinical and the research standpoints, to examine the potential direct association between adolescent social withdrawal and alexithymia, with a view to further clarifying their features, and thereby obtaining more information on the most appropriate types of intervention for the adolescents affected.

### 1.3. The Study: Aims and Hypotheses

Based on the above premises, the main aim of this work was to further analyze the role of alexithymia in adolescent social withdrawal, and the clinical characteristics of the latter. There is still not enough literature on this latter phenomenon, which is becoming increasingly common among teenagers. Despite its growing importance worldwide, there is still no consensus on how to define, diagnose, and manage social withdrawal in adolescence, partly because of the different ways in which it can become manifest and be interpreted in different cultures [8,9,10]. Research conducted to date has focused on social withdrawal mainly as a symptom of a specific diagnostic category, such as depression, social phobia, anxiety disorders, internet dependence, or personality disorders [20,28,29]. There is clearly still a shortage of studies on social withdrawal in its syndromic sense, also considering the issue of managing emotions, i.e., alexithymia. In fact, most studies have analyzed just the behavioral component of this phenomenon, without considering how socially-withdrawn teenagers feel their emotions and their suffering. Currently, due to the lack of research in this field, little is known about the characteristics of socially-withdrawn adolescents, especially from the emotional standpoint. Thus, we decided to better investigate the alexithymic traits of these youths, in order to contribute to the overall understanding of this phenomenon, considering not only its behavioral manifestations, but also the emotional experience.

The first objective of our study was therefore to analyze the link between alexithymia and social withdrawal, also seeking to identify which factor of alexithymia is most impaired in socially withdrawn adolescents. In a sample of adolescents with clinically-diagnosed psychological disorders, we expected to see higher levels of alexithymia in those who were socially withdrawn than in those who were not [29].

As a second objective, we wanted to further examine the adaptability and psycho-behavioral profile of socially-withdrawn adolescents. We predicted that they would be less able to adapt. In terms of the link between social withdrawal and psychopathology, we also expected them to be impaired not only in global functioning, but also and especially in terms of internalizing and social problems [21,22,25,26,27].

In conclusion, the innovative contribution of our work is that it was specifically focused on social withdrawal and its emotional aspects. In addition, it aimed to better investigate the clinical profile of socially-withdrawn adolescents, with the ultimate purpose of developing new trajectories for prevention and treatment programs.

## 2. Methods

### 2.1. Participants and Procedure 

The individuals in our study included patients referred between January 2009 and March 2019 to a semi-residential service for psychopathological disorders in a territorial Neuropsychiatry for Children and Adolescents Unit provided by a local public health service in Padova, Italy. This service offers multidisciplinary intervention for adolescents in situations of psychological, behavioral, and environmental stress, in the form of pedagogical–educational activities, psychotherapy sessions, and pharmacological treatments. When patients first come to the service, the protocol requires that parents sign to give their informed consent to the administration of test materials, and to their use for clinical and research purposes. The present study was part of a broader research project on developmental psychopathologies, conducted in accordance with the Declaration of Helsinki and approved by the local ethical committee (CESC, May 2017, prot.95006).

Questionnaires were completed by the adolescent patients and the educators on the former’s first visit to the semi-residential service. The data used in the present study were collected retrospectively from the patients’ clinical records.

The sample as a whole consisted of 80 patients, including 39 males (48.8%) and 41 females (51.2%). At the time of their referral to the semi-residential service, these patients were from 12 to 17 years old (Average age = 15.2 years, SD = 1.49). As concerns their schooling, they had stopped going to school in 12.5% of cases, while 27.5% and 60% of them were attending lower and upper secondary schools, respectively. The reasons for their neuropsychiatric assessment prior to their referral to the semi-residential program included affective–relational problems (43.8%), behavioral problems (31.2%), and problems at school (25%). Taking the ICD-10 classification system for reference [18], 66.2% of the patients had been diagnosed with an affective–emotional disorder (F30–39, F40–48), while 33.8% had behavioral and personality disorders (F90–98, F60–69).

The power analysis, conducted by means of G*Power 3.1 software, showed that with a sample of 80 participants, we had a power of 0.90 in reliably detecting an odds ratio of 0.31, with a type I error of 0.05 [56].

A case–control study design was chosen to investigate patients’ social withdrawal component. The whole sample was therefore divided into two groups: a group of 40 patients with social withdrawal (Average age = 15.3 years, SD = 1.62), and a group of 40 patients (Average age = 15.2 years, SD = 1.36) matched for age and psychiatric diagnoses with a group without any signs of social withdrawal. For the case group, we first selected all patients who had “withdrawal, isolation, refusal to make contact" among the symptoms recorded on their arrival at the semi-residential service (*n* = 134). This is the format used by the service, which records a patient’s main clinical details and history, obtained from an information sheet completed by the clinician referring the patient to the service. Subsequently, from this initial sample of socially-withdrawn adolescents, we excluded those who did not complete the Toronto Alexithymia Scale-20 (TAS-20), Youth Self-Report 11–18 (YSR), and Global Assessment of Functioning (GAF) on their first visit to the service, or had not a borderline (65–69) or clinical (>69) score on the scale for withdrawal in the YSR (*n* = 94). According to these inclusion and exclusion criteria, the total number of socially withdrawn patients included in our study was 40. The patients excluded from this first step were not placed in the pool to select matched controls. Then, for the control group, we started selecting patients on the basis of their clinical records, in chronological order of their accessing the service. The first inclusion criterion were not having “withdrawal, isolation, refusal to make contact" among the symptoms recorded on their arrival at the semi-residential service, so we automatically excluded all patients who were previously included in the pool to select cases. After this first step, the total number of patients considered was 86. Subsequently, we excluded patients who did not complete TAS-20, YSR, and GAF at the arrival at the semi-residential service or had borderline or clinical scores on the scale for withdrawal in the YSR. The resulting number of patients was 71, of which we selected 40 patients matched for age and psychiatric diagnosis with socially-withdrawn adolescents. Table 1 shows the characteristics of the two groups.

### 2.2. Tools

The validated Italian versions of the following standardized questionnaires were administered:

(a) Global Assessment of Functioning (GAF) [58]: this is a scale compiled by the operators to assess a patient’s psychosocial functioning and activities (at school or at work, interpersonal relations, hobbies, and leisure activities). It considers functioning on a continuum from excellent (100) to severely impaired (1). Individuals are scored and assigned to one of 10 levels, assessing both symptom severity and functional impairment;
(b) Youth Self-Report 11–18 (YSR) [51,59]: this self-reported questionnaire consisting of 113 items that examine social competences and psychopathological behavior. The latter is classified on eight syndrome scales: anxiety–depression; social withdrawal; somatic complaints; social problems; thought disorders; attention disorders; deviant behavior; and aggressive behavior. These subscales are then grouped to obtain three global scales for internalizing problems, externalizing problems, and total problems. In our study, we considered the anxiety–depression, social withdrawal, social problems, internalizing problems, and total problems scales. Items of the anxiety–depression scale reflect symptoms of those syndromes (e.g., fears, worries, sleeping problems, sadness, crying a lot). The social withdrawal scale is made up of items referring to behaviors and individual characteristics (e.g., shyness, isolation, talking difficulties). The social problems scale identifies relational difficulties (e.g., teasing, loneliness, exclusion, clumsiness). The internalizing problems scale is composed of social withdrawal, somatic complaints, and anxiety–depression scales, while the total problems scale is the sum of all the YSR items, reflecting the global level of disease. The tool has a good reliability, with Cronbach’s alpha ranging from 0.71 to 0.95. Specifically, in the present study, Cronbach’s alpha coefficients for each scale ranged from 0.66 to 0.91;
(c) Toronto Alexithymia Scale (TAS-20) [50,60]: this is a self-administered questionnaire comprised of 20 items that respondents score on a five-point Likert scale from “strongly disagree” to “strongly agree”. It consists of three subscales: difficulty describing feelings (DDF; i.e., difficulties in communicating feelings to other people), difficulty identifying feelings (DIF; i.e., problems in recognizing emotions and distinguishing them from bodily sensations), and externally-oriented thinking (EOT; i.e., concrete cognitive style oriented to external reality). Moreover, it has a total score that points out the global level of alexithymia. Respondents obtaining a total score of 61 or more are considered alexithymic, while those who score 50 or less are not alexithymic. A total score between 51 and 60 indicates the possible presence of alexithymia (borderline level). The Italian version of the tool has a good reliability, with Cronbach’s alpha in the range of 0.52 to 0.75 for normal samples, and between 0.54 and 0.82 for clinical samples. In the present study, Cronbach’s alpha coefficients for each scale ranged from 0.50 (for EOT) to 0.78 (for the total score).

### 2.3. Data Analysis

The data were analyzed using Jamovi statistical software, version 1.2 [61].

We first calculated the descriptive statistics and frequency tables to clarify the characteristics of the sample as a whole (*n* = 80) and of the two groups (*n* = 40 each).

Then the chi-square test (*χ*^2^) for categorical variables was used to see whether the two groups were comparable in terms of gender (two levels), age (two levels: 12–14 years and 15–17 years), diagnosis (two levels), and personality organization (three levels).

The *t*-test for independent samples was used to identify any statistically significant differences between the two groups in relation to adaptability, level of alexithymia, and presence of psychopathological features. Social withdrawal (two levels) was input as an independent variable in the model, while the dependent variables were the total score on the GAF, the TAS-20 scales (i.e., DDF, DIF, EOT, and the total score), and several YSR scales, chosen on the basis of the psychopathological issues most often associated with social withdrawal in the literature [21,22,27,28], i.e., anxiety–depression, social problems, internalizing problems, and total problems.

Subsequently, two binomial logistic regressions were run after calculating Pearson’s *r* correlations between the TAS-20 and YSR subscales used as predictors.

The first binomial logistic regression was run to identify the dimension of alexithymia most impaired in socially-withdrawn adolescents. In fact, considering that the presence of alexithymic traits is a characteristic of different psychopathological disorders [43,44,47], we wanted to better investigate which specific aspect of alexithymia (i.e., difficulty in recognizing emotions, difficulty in expressing them, or concrete and externally-oriented thinking) was the most influential on social withdrawal. The presence or absence of social withdrawal (two levels) was input in the model as the dependent variable, with the three subscales of the TAS-20—difficulty describing feelings (DDF), difficulty identifying feelings (DIF) and externally-oriented thinking (EOT)—as predictors.

As mentioned earlier, the literature identifies internalizing problems and social problems as being particularly linked to social withdrawal, so another logistic binomial regression was run to test the direction of this relationship and whether alexithymia influenced it. To be more specific, we were interested in observing whether internalizing problems, social problems, and the global level of alexithymia could significantly predict social withdrawal. Consequently, the presence or absence of social withdrawal was input in the model as the dependent variable, with the scales for internalizing problems and social problems on the YSR, and the total score on the TAS-20 as predictors.

In both binomial logistic regressions, gender, age, diagnosis, and personality organization were input as factors to control for their effects.

Finally, we calculated Pearson’s *r* correlations—considering both the whole sample and cases and controls separately—between the social withdrawal scale of the YSR and (i) the GAF; (ii) the scales for anxiety–depression, social problems, internalizing problems, and total problems in the YSR; and (iii) the scales in the TAS-20.

The level of statistical significance was set at *p* < 0.05.

## 3. Results

### 3.1. Comparability between the Two Groups

No statistically significant association emerged from the *χ*^2^ test between the two groups (with versus without social withdrawal) and gender (*χ*^2^ (1, *n* = 80) = 2.45, *p* = 0.117); age (*χ*^2^ (1, *n* = 80) = 0.238, *p* = 0.626); diagnosis (*χ*^2^ (1, *n* = 80) = 0.503, *p* = 0.478); or personality organization (*χ*^2^ (2, *n* = 80) = 0.468, *p* = 0.791). This means that the variables considered were not influential in defining the groups, which could therefore be compared in terms of the presence or absence of social withdrawal alone.

### 3.2. Social Withdrawal and Alexithymia

The *t*-test for independent samples, with scores on the TAS-20 scales as the dependent variables, identified a statistically significant difference between the two groups on all the scales, with the group of adolescents with social withdrawal always obtaining higher scores (Table 2).

Pearson’s *r* correlations between the TAS-20 scales, calculated before running the binomial logistic regression, are shown in Table 3. They were all positive; more specifically, the correlations between DDF and the other TAS-20 subscales were all moderate, while the correlation between DIF and EOT was small.

Then, from the binomial logistic regression, conducted to see which specific factor of alexithymia was most impaired in the group of socially-withdrawn adolescents, the significant predictor emerged as just the TAS-20 scale for DDF (*β* = 0.26, *z* = 2.84, *p* = 0.005, OR = 1.279, 95% CI: 1.08–1.55). Variables input in the model as factors (i.e., gender, age, diagnosis, and personality organization) did not show any statistically significant effect. McFadden’s *R*^2^ for the model overall was 0.36. 

Lastly, Pearson’s *r* correlations, calculated considering the whole sample, showed that the scale for withdrawal in the YSR correlated significantly and positively with all the TAS-20 scales. Specifically, its strongest associations were with DDF and the TAS-20 total score, while the lowest was with EOT (Table 3). These correlations did not significantly differ considering cases and controls separately (see Appendix A, Table A1).

### 3.3. Social Withdrawal, Adaptability, and Psychological Disorders

When the *t*-test for independent samples was run with the scores on the YSR scales as the dependent variables, statistically significant differences emerged between the groups with and without social withdrawal on all the scales considered (the group with social withdrawal is indicated with the subscript “yes”, while the group without social withdrawal is indicated with the subscript “no”): anxiety–depression (*t*_78_ = 1.45, *p* < 0.001; *M*_yes_ = 73.7, *SE* = 1.76; *M*_no_ = 61.9, *SE* = 1.88); social problems (*t*_78_ = 3.51, *p* < 0.001; *M*_yes_ = 66.3, *SE* = 1.46; *M*_no_ = 59.9, *SE* = 1.11); internalizing problems (*t*_78_ = 6.63, *p* < 0.001; *M*_yes_ = 72.8, *SE* = 1.11; *M*_no_ = 58.9, *SE* = 1.79); and total problems (*t*_78_ = 4.21, *p* < 0.001, *M*_yes_ = 67.8, *SE* = 1.21; *M*_no_ = 58.8, *SE* = 1.77). The group with social withdrawal always scored higher. No significant differences emerged for the GAF.

Finally, Pearson’s *r* correlations, calculated considering the whole sample, showed statistically significant associations between the scale for social withdrawal and all the other scales considered in the YSR (Table 4). The correlations were all positive and strong, ranging from 0.52 (with social problems) to 0.74 (with internalizing problems). The correlation with the score in the GAF was also significant, but low and negative. These correlations did not significantly differ when considering cases and controls separately (see Appendix A, Table A1).

### 3.4. Predictors of Social Withdrawal in Adolescents

Pearson’s *r* correlations between the predictors included in the binomial logistic regression (i.e., the TAS-20 total score, as well as the social problems and internalizing problems scales of the YSR) were all statistically significant, positive, and strong (Table 5).

Subsequently, from the binomial logistic regression, it emerged that the predictors of social withdrawal in the model were the scores for internalizing problems on the YSR (*β* = 0.16, *z* = 2.89, *p* = 0.004, OR = 1.18, 95% CI: 1.05–1.32) and the total scores on the TAS-20 (*β* = 0.11, *z* = 2.42, *p* = 0.015, OR = 1.12, 95% CI: 1.02–1.23), while social problems were not significant. Moreover, gender was also a significant factor in the model (*β* = −2.17, *z* = −2.41, *p* = 0.016, OR = 0.11, 95% CI: 0.02–0.67), with a higher probability (*Pr*) for males (*Pr* = 0.04) of being in the group of socially-withdrawn adolescents compared to females (*Pr* = 0.006). The McFadden *R*^2^ for the overall model was 0.48.

## 4. Discussion

Our first aim in this study was to investigate the direct relationship between social withdrawal and alexithymia. Significant differences emerged between our two groups with and without social withdrawal in every dimension of alexithymia, with socially-withdrawn adolescents scoring higher on all the scales. Our findings thus confirm that socially-withdrawn adolescents are more impaired in terms of emotional competence, expanding on what emerged from the study by Hattori [2] on emotional problems in socially-withdrawn individuals. Our data also show that difficulty describing feelings (DDF) is the only alexithymia factor influential on social withdrawal. Moreover, DDF is the TAS-20 subscale most correlated to the social withdrawal scale on the YSR. In other words, socially-withdrawn adolescents have a general difficulty with managing their emotions, and are specifically poor at communicating their feelings. This finding confirms Piotti’s report, based on clinical experience, that socially-withdrawn adolescents find it difficult to put their feelings and experiences of suffering into words [52].

Some published studies regarding adolescents have associated alexithymia with various psychopathological conditions [43,44,47], because it has been found to be related to an emotional dysregulation acquired already in infancy, and transmissible from one generation to the next [34,35,36]. The adolescents in the present study had a psychiatric diagnosis with which alexithymia may be associated. Judging from the mean total scores in the TAS-20, the individuals in our group with social withdrawal were alexithymic, while those in the group without social withdrawal were borderline for alexithymia. This would indicate that our whole sample had emotional problems, but what distinguished the socially withdrawn was a greater difficulty in verbalizing their suffering. The inability to ask for help may induce these adolescents to identify withdrawal within their own private worlds and self-exclusion from any form of social interaction as the only strategies able to alleviate their underlying discomfort.

As for the second aim of this study, to establish the psycho-behavioral profile of socially-withdrawn adolescents, our data confirm the reports of an association between social withdrawal and internalizing problems, including anxiety–depression and social problems [22]. In fact, our group with social withdrawal scored significantly higher on the YSR scales measuring these constructs, as well as for total problems. In other words, they showed a greater degree of psycho-emotional suffering, even though there was no significant difference between the two groups with regard to global functioning (GAF). This would suggest that social withdrawal does not compound the contribution of other psychopathological elements to an individual’s social, occupational, and psychological functioning in response to the various problems encountered in life.

Since several published studies have considered the relationship between internalizing problems, social problems, and social withdrawal, we tried to examine the direction of this relationship, and to investigate the role of alexithymia. Our data indicate that internalizing problems and alexithymia have a bearing on social withdrawal, while social problems did not emerge as a significant predictor of this type of behavior. We surmise that difficulties with managing emotions (and particularly with communicating them), and internalizing symptoms may be antecedents of social withdrawal, and risk factors for its onset. Social problems, on the other hand, could develop as a consequence of social withdrawal, further trapping the adolescent in a vicious cycle of exclusion from relationships. Another possible explanation is that social problems are not directly linked to social withdrawal, but they may precede internalizing problems. These in turn, along with the presence of alexithymic traits, may encourage withdrawal behaviors.

Moreover, gender also has a significant effect in the model, with a higher probability for males to be in the group of socially-withdrawn adolescents. This would indicate that males with internalizing problems and alexithymia are more at risk of developing social withdrawal compared to females with the same difficulties. Previous studies have shown a higher prevalence of socially-withdrawn adolescents among males [2,3,28]. Our findings thus are in line with those in the literature, and add information about the individual characteristics of boys at risk for withdrawal. Nevertheless, the relationship between gender and social withdrawal should be better investigated in future research.

Although the findings of this study are intriguing, there are some limitations that need to be mentioned, which could also serve as a starting point for future studies. First of all, there is the relatively small size of our sample, and the fact that it only included adolescents in northern Italy. Moreover, another limitation is linked to the retrospective nature of the investigation, which did not allow us to obtain all the information of interest for some participants. Then there is the fact that we used self-report questionnaires: although they have demonstrated their clinical value and are quick to administer, they can suffer from a bias relating to social desirability. Subsequently, the EOT scale of the TAS-20 showed a low reliability in our sample (*α* = 0.50). This result is consistent with previous studies with adolescents (e.g., [46,62,63,64,65]), which have reported low Cronbach’s alpha values for such scales, ranging from 0.29 to 0.56. Consequently, the EOT scale seems problematic in the adolescent population in general, and not just in our sample. A possible explanation is that externally-oriented thinking does not represent a core feature of alexithymia in adolescents, but it may be more suitable just for adults. La Ferlita et al. [64] stated that this difference between adults and adolescents might be linked to the different strategies used to face emotional difficulties, according to the specific developmental age. Nevertheless, considering that the TAS-20 is widely used, further studies are needed to better investigate the factorial structure of the overall measure and the reliability of the EOT scale in community and clinical adolescent samples. Another shortcoming of the present study is that we considered social withdrawal as a unidimensional construct, so we did not analyze the different components of the phenomenon. This certainly is a limitation of the present study, but it may also be a starting point for future research. In fact, it would be useful to better investigate such a construct on the basis of Asendorpf’s model [13,14]. Furthermore, future studies should include other individual and social variables that may constitute risk factors for adolescent social withdrawal (e.g., attachment, relationships with peers and family, specific traumas, social support). Lastly, since the association between social problems and social withdrawal is still not clear, it would be interesting to deepen the knowledge of both this relationship and the role that variables involved play in it.

In conclusion, even with these limitations, our research adds a novel contribution to the still-limited literature on social withdrawal in adolescence. Our focus on the alexithymic traits of socially-withdrawn adolescents could be particularly helpful for the purposes of treatment and prevention. Intervention to improve these teenagers’ emotional competence could be a useful way to help them verbalize their psycho-emotional unease, and find more functional ways to deal with it. Understanding the features of social withdrawal in adolescence may be important in the scholastic context, too. In fact, one of the early signs of possible withdrawal is dropping out of school, which represents a risk for adolescents’ mental health. Therefore, teachers should be able to recognize adolescents at risk in advance, in order to avoid negative outcomes. Knowledge about the alexithymic traits of socially-withdrawn adolescents could be relevant for both teachers and clinicians. In fact, they could cooperate in the implementation of prevention programs at school, with activities based on students’ emotions, dialing in on the best strategies to turn those emotions into words.

Given the increasing frequency with which we are seeing social withdrawal in adolescence, it is hugely important to develop ways to prevent it in order to reduce the risks inherent both in social withdrawal per se, and in the psychopathological conditions that may be associated with it.

## Figures and Tables

**Table 1:** Frequencies of the characteristics of the case and control groups.

		Group	
		Cases *n* (%)	Controls *n* (%)
Sex	Males	23 (57.5%)	16 (40.0%)
	Females	17 (42.5%)	24 (60.0%)
Age	12–14 years old	11 (27.5%)	13 (32.5%)
	15–17 years old	29 (72.5%)	27 (67.5%)
Personality organization ^1^	Neurotic	11 (27.5%)	9 (22.5%)
	Borderline	22 (55%)	25 (62.5%)
	Psychotic	7 (17.5%)	6 (15.0%)
Diagnosis ^2^	F30–39, F40–48	25 (62.5%)	28 (70.0%)
	F90–98, F60–69	15 (37.5%)	12 (30.0%)
Traumas	Yes ^3^	31 (77.5)	26 (65.0%)
	No	9 (22.5%)	14 (35.0%)
Attendance at the semi-residential service	Continuous	35 (87.5%)	31 (77.5%)
	Discontinuous	5 (12.5%)	9 (22.5%)
Hours per week of attendance at the semi-residential service	1–5	3 (7.5%)	2 (5.0%)
	5–10	12(30.0%)	23 (57.5%)
	10–15	14 (35.0%)	10 (25.0%)
	>15	11 (27.5%)	5 (12.5%)
Parental couple	Single parent ^4^	14 (35.0%)	16 (40.0%)
	Intact	26 (65.0%)	24 (60.0%)
Parents’ education level ^5^	High	11 (27.5%)	6 (15.0%)
	Average	20 (50.0%)	23 (57.5%)
	Low	9 (22.5%)	9 (22.5%)
	Not known	0	2 (5.0%)

Notes: ^1^ Personality organization as established from a structural interview based on Kernberg’s criteria [57]. ^2^ F30–39, F40–48 = affective–-emotional disorders; F90–98, F60–69 = behavioral and personality disorders. ^3^ In this category are included the following typologies of trauma, reported in the format used by the semi-residential service: conflict/domestic violence, separation/deaths, psychiatric disorders, changes/relocations. ^4^ Single parent due to separation or death of the other. ^5^ Parents’ education level: high = both parents have a university degree, or one has a degree and the other a high school diploma; average = both parents have a high school diploma, or one has a degree and the other completed middle school; low = both parents completed middle school, or one has a high school diploma and the other completed middle school.

**Table 2:** Means and results of the *t*-tests, with scores in the Toronto Alexithymia Scale (TAS-20) as the dependent variables and group (with vs without social withdrawal) as the independent variable.

TAS-20 Scales	Group	*M* (*SE*)	*t*	*df*	*p*
DDF	with social withdrawal (*n* = 40)	18.0 (0.59)	5.43	78	<0.001
	without social withdrawal (*n* = 40)	13.3 (0.65)			
DIF	with social withdrawal (*n* = 40)	23.0 (1.03)	4.00	78	<0.001
	without social withdrawal (*n* = 40)	16.9 (1.13)			
EOT	with social withdrawal (*n* = 40)	23.6 (0.69)	2.36	78	0.021
	without social withdrawal (*n* = 40)	21.4 (0.63)			
TOT	with social withdrawal (*n* = 40)	64.7 (1.57)	5.65	78	<0.001
	without social withdrawal (*n* = 40)	51.6 (1.69)			

Notes: DDF = difficulty describing feelings; DIF = difficulty identifying feelings; EOT = externally-oriented thinking; TOT = total score; *M* = mean; *SE* = standard error.

**Table 3:** Pearson’s *r* correlations between the TAS-20 values and the social withdrawal scale of the Youth Self-Report 11–18 (YSR), considering the sample as a whole.

	DDF	DIF	EOT	TOT
DDF	-	0.47	0.36	0.79
DIF	0.47	-	0.13	0.83
EOT	0.36	0.13	-	0.57
Withdrawal	0.59	0.40	0.38	0.60

Notes: DDF = difficulty describing feelings; DIF = difficulty identifying feelings; EOT = externally-oriented thinking; TOT = total score of the TAS-20; Withdrawal = social withdrawal scale of the YSR.

**Table 4:** Pearson’s *r* correlations between the social withdrawal scale of the YSR, the other YSR scales, and the Global Assessment of Functioning (GAF), considering the sample as a whole.

	Anx-Dep	Int. Prob	Soc. Prob	YSR TOT	GAF
Withdrawal	0.62	0.74	0.52	0.56	–0.21

Notes: Withdrawal = social withdrawal scale; Anx-Dep = anxiety–depression scale; Int. Prob = internalizing problems scale; Soc. Prob = social problems scale; YSR TOT = total problems scale.

**Table 5:** Pearson’s *r* correlations between the TAS-20 total score and the social problems and internalizing problems scales of the YSR.

	Soc. Prob	Int. Prob	TAS TOT
Soc. Prob	-	0.66	0.46
Int. Prob	0.66	-	0.63

Notes: Soc. Prob = social problems scale; Int. Prob = internalizing problems scale; TAS TOT = total score of the TAS-20.

## Data Availability

The data presented in this study are available on request from the corresponding author. The data are not publicly available because they report private information about participants.

## Appendix A

**Table A1:** Pearson’s *r* correlations between the social withdrawal scale of the YSR and the other scales of the YSR, the TAS-20, and the GAF, considering cases and controls separately.

	Group	
	Cases Withdrawal Scale	Controls Withdrawal Scale
DDF	0.33	0.31
DIF	0.07	0.15
EOT	0.33	0.22
TAS TOT	0.31	0.30
Anx-Dep	0.47	0.55
Soc. Prob	0.33	0.50
Int. Prob	0.57	0.66
YSR TOT	0.36	0.55
GAF	−0.20	−0.19

Notes: Withdrawal = social withdrawal scale; Anx-Dep = anxiety-depression scale; Soc. Prob = social problems scale; Int. Prob = internalizing problems scale; YSR TOT = total problems scale of the YSR; GAF = Global Assessment of Functioning; DDF = difficulty describing feelings; DIF = difficulty identifying feelings; EOT = externally-oriented thinking; TAS TOT = total score of the TAS-20.
